# Automatic mouse ultrasound detector (A-MUD): A new tool for processing rodent vocalizations

**DOI:** 10.1371/journal.pone.0181200

**Published:** 2017-07-20

**Authors:** Sarah M. Zala, Doris Reitschmidt, Anton Noll, Peter Balazs, Dustin J. Penn

**Affiliations:** 1 Konrad Lorenz Institute of Ethology, Department of Integrative Biology and Evolution, University of Veterinary Medicine, Vienna, Austria; 2 Acoustic Research Institute, Austrian Academy of Sciences, Vienna, Austria; Texas Christian University, UNITED STATES

## Abstract

House mice *(Mus musculus)* emit complex ultrasonic vocalizations (USVs) during social and sexual interactions, which have features similar to bird song (i.e., they are composed of several different types of syllables, uttered in succession over time to form a pattern of sequences). Manually processing complex vocalization data is time-consuming and potentially subjective, and therefore, we developed an algorithm that automatically detects mouse ultrasonic vocalizations (*Automatic Mouse Ultrasound Detector* or A-MUD). A-MUD is a script that runs on STx acoustic software (S_TOOLS-STx version 4.2.2), which is free for scientific use. This algorithm improved the efficiency of processing USV files, as it was 4–12 times faster than manual segmentation, depending upon the size of the file. We evaluated A-MUD error rates using manually segmented sound files as a ‘gold standard’ reference, and compared them to a commercially available program. A-MUD had lower error rates than the commercial software, as it detected significantly more correct positives, and fewer false positives and false negatives. The errors generated by A-MUD were mainly false negatives, rather than false positives. This study is the first to systematically compare error rates for automatic ultrasonic vocalization detection methods, and A-MUD and subsequent versions will be made available for the scientific community.

## Introduction

House mice *(Mus musculus)* emit a wide repertoire of vocalizations across five octaves (from ca. 3 to more than 120 kHz), which are mostly ultrasonic vocalizations (USVs), beyond the range of human hearing (>20 kHz). Mice begin emitting USVs as pups, which function as ‘isolation calls’ that induce maternal retrieval [1], and adults of both sexes produce complex USVs during courtship and other social contexts (see reviews [2, 3–9]). Adult males, for example, emit USVs when presented with a female mouse, or their scent, and during courtship and copulation (laboratory mice [10–16]; wild house mice [17]). Vocal production is generated by a ‘glottal jet’ mechanism [18], and the USVs of male mice are innate since they do not use [19] or require [20, 21] auditory feedback (vocal learning). On the other hand, females acquire their auditory preferences for the USVs of unrelated males [17] at an early age, through auditory learning (familial imprinting) [22]. USV emission is under neuro-endocrine control [2, 9], and varies among individuals depending upon age, sex, genetic background, social status, health, and other factors [2, 8, 23–25]. Individuals can modulate USV emission during social and sexual interactions, depending upon their motivation or behavioral state, target receiver, and other contexts [8]. The signals transmitted in USVs (‘information content’, ‘meaning’ or ‘semantics’) and other possible functions are not well understood, though playback experiments with wild house mice indicate that they are sufficient for mice to recognize and discriminate *Mus* species, close kin, and individuals [17, 26, 27]. There are still few studies on wild house mice [25, 28–31], however, and none in natural or naturalistic ecological or social conditions. Most USV studies have focused on laboratory mice (*Mus laboratorius)* [32], which are used as a model system to investigate the genetic basis of communication and disease phenotypes [33], including human speech, autism and other neuropsychiatric disorders [4, 5]. Mouse vocalizations may also provide useful non-invasive indicators of disease and animal welfare [34–36]. Thus, research on mouse USVs is increasing, and this work has largely been inspired by the discovery of their complexity and similarity to birdsong [15], which was made possible by technical developments in bioacoustic analyses.

Mouse USVs are structurally and temporally complex at multiple levels of acoustic analyses [8]. First, USVs can been classified into different types of *syllables*, which consist of single notes (pure tones) or complex syllables with frequency jumps, and can be classified according to their particular spectrotemporal features, i.e., amplitude, frequency and duration (‘syllable acoustics’). Mice emit as many as 12 different types of syllables, and the number (‘vocal repertoire’) and the frequency in the usage of different syllables (‘prevalence’) can vary within and among mice. Syllable classifications vary among researchers [8, 15, 29, 37–40], and the challenge is to determine whether or how mice perceive and respond to different syllables. Laboratory mice can discriminate at least four types based on their ability to be trained by reinforcement (operant conditioning) [41]. Second, mice vary the number of syllables emitted per minute (‘vocalization rate’). Males emit around 13 to 90 syllables/min upon presentation of a female urine stimulus (in wild mice [29] or laboratory mice [42], respectively), and laboratory mice produce up to 160 to 230 syllables/min when presented with an anesthetized mouse or an intruder [43]. Third, mice emit USV syllables in phrases or bouts, composed of a series of syllables, usually more than one type, in succession over time to form a pattern of sequences [15]. The sequence of different syllables can vary (‘syntax’), as well as the number and type of different bouts (Markovian chains) [30, 31]. Thus, there is a surprisingly amount of complexity in mouse USVs, and better and more efficient methods for data processing and analyzing are needed to improve our understanding of these vocalizations.

Developing methods for automatically detecting and classifying types of USVs (syllables) presents a difficult technical challenge, especially for large sound files and recordings of mice in social conditions. Previous studies have usually relied on manually counting the syllables in spectrograms, which is time intensive (e.g., [17, 24, 28, 39, 42]). Other studies have used an in-house script for automated or semi-automated detection (e.g., [15, 30]); or used commercial software to process recordings automatically or semi-automatically, e.g., *Avisoft SAS LabPro* (Avisoft Bioacoustics, Germany) [20, 38, 43] and *Sound Analysis Pro* (SAP) [26, 29]. The error rates of these automated detection methods have never been evaluated to our knowledge, aside from a short paragraph in one study [15], and concerns have been raised about their reliability [17, 24, 28, 30, 39, 42, 44]. One of the main challenges of developing automatic detection methods is that USVs have a very low signal-to-noise ratio (SNR), and broadband interference from ambient, background noise (i.e., sounds other than previously described mouse USVs), which can generate false positives or mask the signals (false negatives). We developed an algorithm to automatically detect USV syllables (*Automatic Mouse Ultrasound Detector* or A-MUD), which is a script run in *STx*, S_TOOLS-STx (Acoustic Research Institute, Austria). We measured the error rates of A-MUD using manually segmented files as a ‘gold standard’ reference, and we compared its performance with a commercially available program. This first version of A-MUD is a step towards improving error rates and developing automated syllable classification, and our aim is to provide a tool that will help researchers improve the efficiency of their analyses.

## Materials and methods

### Subjects and housing

Our study was conducted with wild-derived house mice (*Mus musculus musculus*), the F1 offspring of wild mice caught at the Konrad Lorenz Institute of Ethology (48°12’38”N, 16°16’54”E) in Vienna, Austria. Previous studies on this subspecies were conducted only on male USVs elicited by urinary odors, and female responses to male USV playbacks [2, 17, 26–29]. Mice were raised in mixed-sex family groups (standard Type IIL cages, 36.5 x 20 x 14 cm, with stainless steel cover, 1cm mesh width, Tecniplast, Germany) until weaning (21 d of age). Siblings were housed in mixed-sex groups (maximum of four mice per cage) until 5 weeks of age, when the sexes got separated. Males were individually housed to prevent fighting and females were housed in sister pairs. All cages were provided equally with wood shavings (ABEDD, Austria), nesting material (Nestlet, Ehret, Austria), one cardboard paper roll and one nest box (Tecniplast, Germany) for environmental enrichment. Food (rodent diet 1324, Altromin, Germany) and water were provided *ad libitum*. Mice were kept in standard conditions (mean±SD room temperate: 22 ± 2°C, in a 12:12 h light:dark cycle, lights off at 15:00). Red light was used instead of a complete dark period to be able to conduct experiments during the mice active period without disturbing them. We worked with 32 adult mice (mean±SD age: 192±19d; n = 11 males, n = 21 females). We did not sacrifice any of the mice used for this study.

### Recording apparatus

We recorded the vocalizations of males (n = 11) and females (n = 3) (‘callers’) in the presence of a female (‘stimulus’) under red light, during the active period of the day for our mice (15:00 to 17:30). The callers were primed (or socially experienced) for 5 min 1 d prior to the recordings by placing a female into their home cage. Some stimulus females were used once as priming animals and once as stimuli, but never for the same caller. The callers were always unfamiliar and unrelated to the priming and stimulus females. The recording apparatus consisted of a Plexiglas cage (36.5 x 21 x 15 cm) divided into two equal compartments, the ‘caller’ and the ‘stimulus compartments’. To ensure that the mice could see and smell each other during the experiment, the two compartments were separated by a 0.5 cm thick Plexiglas divider covered with small holes (0.5 cm diameter). The caller compartment was covered with a metal cage lid (1 cm width mesh), whereas the stimulus compartment was covered with a Plexiglas lid to prevent USVs from being recorded. This design ensured that we recorded vocalizations of the caller (the focal mouse), and not the stimulus mouse, and preliminary tests using USVs playbacks released from an ultrasound speaker (Avisoft Bioacoustics, Germany) positioned into the stimulus compartment, confirmed that the Plexiglas cover was very effective at blocking USVs. The stimulus compartment was also provided with bedding and 2–3 food pellets.

We always used a small plastic cylinder to introduce the mice into their respective compartments. To record, we first placed the stimulus female into the assigned compartment and after 5–10 min habituation time we introduced the focal mouse. The entire cage was then positioned inside a recording chamber, which was lined with acoustic foam as described in [27]. A condenser ultrasound microphone (Avisoft Bioacoustics/CM16/CMPA with an integrated pre-amplifier and a frequency range from 10 to 200 kHz) and an UltraSoundGate 116–200 (Avisoft Bioacoustics, Germany) were mounted inside the recording chamber, 10 cm above the caller compartment. Before each recording, the microphone was calibrated with a 440 Hz tone of a commercial available tuning fork. Mice were recorded using the RECORDER USGH software with settings at 300 kHz sampling rate, 16 bit format, and 256 Hz FFT size. After positioning the cage inside the recording chamber, we waited for 30 sec and then started recording for 10 min. To avoid and also to standardize any potential estrus status effects of the stimulus female on the caller, we added an additional olfactory stimulus (5 μl of 4 different pooled female urine on a 4 x 4 cm filter paper) into the caller’s compartment. The urine was previously collected in metabolic cages (Techniplast, 600M021) from wild-caught adult females, equally aliquoted and mixed in Eppendorf tubes and stored at -20°C until the recordings. After each recording the entire cage was cleaned with ethanol before reusing.

### Development and implementation of the *Automatic Mouse Ultrasound Detector* (A-MUD)

We implemented a segmentation algorithm in a new script (*Automatic Mouse Ultrasound Detector* or A-MUD 1.0) in STx (S_TOOLS-STx version 4.2.2), a software from the Acoustic Research Institute (Austria), which is free for scientific use (http://www.kfs.oeaw.ac.at/stx). STx is used for processing large quantities of data in a timely fashion [45, 46], such as for speech analysis [47, 48], noise evaluation [49, 50], and psychoacoustics [51]. It is designed to organize and process large collections of signal and segment data and to implement a large number of signal processing algorithms and elaborate interactive tools. STx has been used for bio-acoustic projects [52], particularly for signal detection and segmentation. It provides a scripting language for extending the software for problem-specific applications. The scripting language can access all the program interfaces (e.g., graphic, file system), the signal and segment data and the extensive internal signal-processing functions. In addition, STx can be extended using C/C++ functions, where it is possible to perform complex methods in a timely fashion. Moreover, STx provides all the necessary functions and tools to interactively check and correct the automatically generated segments, and it also includes flexible and programmable export methods to facilitate segment analysis in other programs, such as R or Microsoft Excel.

#### Development of A-MUD and its segmentation algorithm

As previously mentioned, one of the main challenges of automatically detecting USVs is the very low signal-to-noise ratio (SNR), as USV signals often have broadband interference or ambient noise, which can partially mask the signals. Mouse USVs occur between 20–120 kHz, and are nearly mono-frequency signals, similar to a whistle. They have a narrow bandwidth, and modulate over a relatively large frequency range, and their duration is 5–100 ms (though some researchers also include < 5 ms sounds, e.g., [38, 39, 44]). Here, we propose a new method that can cope with low SNR. To segment the signal and separate the USV from the background, the script uses the narrow bandwidth, which is a characteristic property not shared by the other part of the recorded signal. All the necessary calculations for the signal detection are done in the time-frequency domain. A short-time Fourier transformation (STFT) [53, 54] with window *w* of length *N*_*win*_ is applied to the signal *f* with hop size H and FFT-length *N*_*FFT*_:

$$STFT_{w}(f)[k,l]=\sum_{j=0}^{N_{FFT}-1}f[j]\cdot w[j-kH]\cdot e^{-2\;\pi i\;(j-l)l\;N_{FFT}}$$

A Hanning window was chosen for this task. The high sampling rate (250 or 300 kHz) and the shortness and frequency dynamics of the signal require the window length to be between 2.5 and 3.3 ms (choosing *N*_*FFT*_ = *N*_*win*_ this leads to a frequency resolution being *Δf* = 300–400 Hz) and an overlap of between 75% and 85% (hop size *H* ~ 0.5 ms), which was determined empirically to provide a good time-frequency representation of the USVs. All further calculations are restricted to the power spectra (PS) in the relevant frequency range between 30–120 kHz, assumed to result in N frequency bins. We performed following three steps:

**Step 1: Noise reduction (pre-whitening).** For de-noising we used a pre-whitening step (see e.g., [55]). The signal energy (*rms*) of the power spectra was calculated for each frame. As an estimation of the maximum level of the background noise, we set L_*noise*_ = L_*95*_ + 2dB, where L_*95*_ is the 95% quantile of all *rms* values. The background noise spectrum was estimated by averaging the spectra of all frames with a level less than L_*noise*_. All power spectra were then multiplied with the inverse background spectrum, for the removal of stationary noise. This procedure of pre-whitening has great effects when applied in methods using amplitude as a criterion [56].

**Step 2: Compute the segmentation parameter track and set thresholds.** The narrowness of the signal's bandwidth (*ebw*) used for segmentation, was defined as the number of frequency bins for which a certain ratio *R*_*ebw*_ of the total spectrum energy was achieved, i.e., *ebw* is the smallest number for which

$$R_{ebw}\cdot \sum_{i=1}^{N}PS_{i}\leq \sum_{i=1}^{ebw}PS_{i}.$$

Here, the power spectrum is sorted by descending amplitude. This is done for every time step. This ratio was chosen between 0.3 < *R*_*ebw*_ < 0.6. The inverse of *ebw* is used as the main segmentation criterion. We define the energy concentration by $ec=\frac{N}{ebw}$. Two threshold values for the segmentation algorithm are calculated using the *ec* function: *ec*_*on*_ = 10% quantile of the *ec* function and *ec*_*off*_ = 90% quantile of the *ec* function. Windows with high *ec* can be considered as containing a signal and those with low *ec* as noise.

**Step 3: Detect segments and apply time corrections.** The *ec* function is then used to detect segments. The *ec* function is searched for a local maximum greater than the *ec*_*on*_ threshold. The *ec* function is then applied from this position forward and backwards, and until a point where the *ec* value falls below the *ec*_*off*_ threshold. These points are used as the beginning and end positions of the segment. This procedure is repeated until no more matching local maxima are found.

Two correctional steps are applied to the identified segments. First, any sequential segments closer than the minimal distance *t*_*mindist*_ (5–10 ms) are merged. Second, any segments shorter than the set minimum length *t*_*mindur*_ (5–10 ms) or longer than the maximum length *t*_*maxdur*_ (150–200 ms) are removed. The temporal thresholds *t*_*mindist*_, *t*_*mindur*_ and *t*_*maxdur*_, and all other parameters of the algorithm are derived from a heuristic pre-test and analysis of a small set of manually segmented test signals. The method was then evaluated using a much larger signal set.

#### Implementing and testing A-MUD

Although the USV detection algorithm is straightforward, it requires high computational effort due to the high sampling rate (up to 300 kHz) and the overlapping necessary for the short length of the calls. The script automatically detects USVs in the recorded sound file. In addition, A-MUD also provides the spectrographic analyses of each detected *element* (i.e., these are candidate syllables) providing its frequency, amplitude and time parameters (Table 1). The resulting segments and their extracted frequency progression parameters are stored as signal metadata in an XML-file. Computation is taking 1.5 to 2.5 times longer than the signal (file) length.

**Table 1:** USV parameters and definitions in A-MUD.

Parameter	Definition of the parameter (measurement unit)
**begin**	start of the element (s)
**length**	length of the element (s)
**fmean**	mean frequency of the element (Hz)
**fband**	frequency bandwidth (fmax—fmin) (Hz)
**amean**	mean amplitude of the element (dB)
**t1**	start point of the element (ms)
**f1**	frequency at start point of the element (Hz)
**a1**	amplitude at start point of the element (dB)
**tn**	end point of the element (ms)
**fn**	frequency at endpoint of the element (Hz)
**an**	amplitude at endpoint of the element (dB)
**tfmin**	time point of lowest frequency of the element (ms)
**fmin**	lowest frequency of the element (Hz)
**afmin**	amplitude at the point of lowest frequency (dB)
**tfmax**	time point of highest frequency of the element (ms)

Spectrographic parameters detected by A-MUD.

Initially, recordings from four male callers were used to develop the A-MUD algorithm and to choose the appropriate values for the given parameters. We then evaluated error rates of A-MUD and a commercially available software using 10 additional recordings (7 male and 3 female callers). The 10 recordings cover the range of calling rates typical for wild-derived mice ([17, 27, 29]; Zala et al. unpublished data). These 10 recordings were manually segmented three different times to obtain a gold standard reference (see below). We used the gold standard reference first to assess the inter-observer reliability within STx and the intra-observer reliability between both programs, and then to estimate the error rates of the two automatic call detection methods. Thus, two independent observers manually segmented the files in STx, and in addition one manual segmentation was performed in the commercially available software (same observer for both programs). The observers marked the beginning and the end of each syllable and classified 12 different syllable types depending on their duration, frequency and frequency modulation, according to previous classification [27, 29, 40]. We then compared the error rates of A-MUD and the commercially available program. The automatic analysis in the commercially available software was conducted using two different settings. First, using the setting ‘whistle tracking’, which is based on detecting steady signals without rapid frequency modulations and is recommended for analyzing soft whistle like sounds in noisy background such as short syllables of rodent USVs. Second, using the setting ‘single threshold’, which detects all elements above a specified amplitude threshold.

Each of the 10 sound files was thus processed six times using both programs and as follows: (1) commercially available software, manual observer 1 (n = 10), (2) commercially available software, setting ‘whistle tracking’ (n = 10), (3) commercially available software, setting ‘single threshold’ (n = 10), (4) STx, manual observer 1 (n = 10), (5) STx, manual observer 2 (n = 10), (6) STx, A-MUD (n = 10). The settings chosen to create the spectrograms for manual segmentation were the best time and frequency resolutions for visually analyzing mouse USVs in both programs. The spectrograms for the manual segmentations in the commercially available software were generated using following settings: FFT length = 512, frame size = 100% (flat top window) and overlap = 50%. The settings of the automatic processing in the commercially available software (single threshold) were: threshold = -50 dB and hold time = 20 ms; and the settings for the same software (whistle tracking) were: tolerated maximum change of frequency modulation 10 253 Hz, hold time = 20 ms. For all automatic analyses in the commercially available software only frequencies > 25 kHz and only elements ≥ 10 ms were included. All the settings were empirically chosen, to maximize the quality of USV detection. For the manual segmentation in STx we scrolled through the whole spectrogram in steps of 2 seconds with the overlap of 25%. Spectrograms were generated with a range of 50 dB, frame = 4 ms and an overlap of 75%. The spectrograms used a Hanning window and displayed frequencies between 25–150 kHz. For automatic processing in STx, we ran the script A-MUD 1.0. This script’s threshold of element duration was set at 10 ms.

#### Calculating error rates

We first confirmed the reliability of our manual segmentation by comparing the total number of manually detected elements between individual observers and within the same observer using the two detection programs (as their spectrograms present visually different patterns). After confirming the reliability of our manual detection (see Results), we used these manually segmented data as our gold standard reference to calculate the percentage of elements that were detected correctly (correct positives, ‘correct’), missed incorrectly (‘false negatives’), and detected incorrectly (‘false positives’) by the two automatic call detection programs. Thus, we compared the ‘manual reference elements’ (Nref) with the ‘corresponding elements*’* (Ncorr). Nref was calculated using only the elements that were detected by all three manual segmentations, i.e. the gold standard. Ncorr identified the elements that were detected by both the manual (Nref) and the automatic processing methods (Nauto) within each software separately. We used following formulas:

$$\begin{array}{l} Correct=\left(100*\mathrm{Ncorr}\right)/\mathrm{Nauto}\\ False\;positives=\left[100*\left(\mathrm{Nauto}–\mathrm{Ncorr}\right)\right]/\mathrm{Nauto}\\ False\;negatives=\left[100*\left(\mathrm{Nref}–\mathrm{Ncorr}\right)\right]/\mathrm{Nref} \end{array}$$

**Fig 1:**
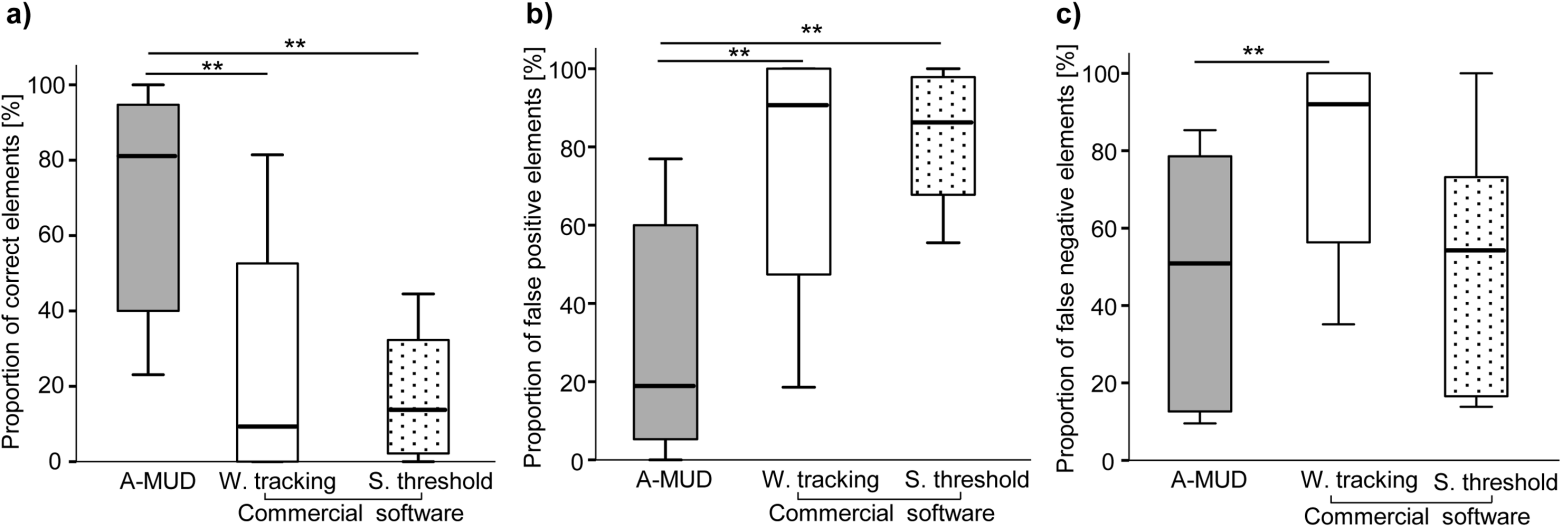
Evaluation of error rates among automatic USV detection methods (all recordings). Boxplots showing the percentage of correct positives (a), false positives (b) and false negatives (c) comparing three automatic processing methods: A-MUD (grey) and a commercially available software using ‘whistle tracking’ (white) or ‘single threshold’ (stippled) settings. The graph shows median ± 95% CI, including the 25th and the 75th percentiles. ** = p ≤ 0.01.

**Fig 2:**
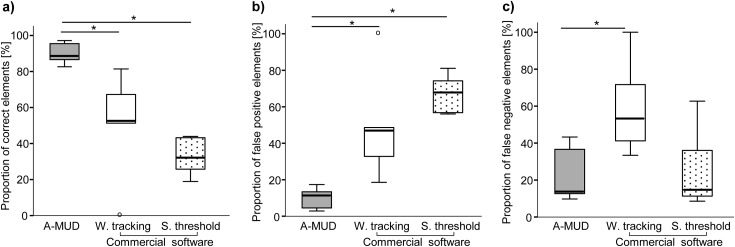
Evaluation of error rates among automatic USV detection methods (selected recordings). Boxplots showing the percentage of correct positives (a), false positives (b) and false negatives (c) with three automatic processing methods: A-MUD (grey) and a commercially available software using ‘whistle tracking’ (white) or ‘single threshold’ (stippled) settings. The graph shows median ± 95% CI, including the 25th and the 75th percentiles. * = p ≤ 0.05. ° = outliers.

Error estimates were calculated for all 10 files first by using the entire manually segmented dataset Nref, which also contained short USV elements < 10 ms (Fig 1), and then again after excluding these short elements from the gold standard (Fig 2), and here we explain the reason for this second analysis. Short sounds in USV recordings pose a technical challenge for automatic (and sometimes even manual) detection because they are very difficult to distinguish from background noise. We therefore set a threshold in A-MUD and in the commercial software to detect only sounds ≥10 ms, as described above, to reduce the number of false positives [6]. The downside of this threshold is that both methods will fail to detect very short elements that mice emit (false negatives). This is an acceptable tradeoff [57], as long as most of these short sounds are background noise and not USVs. However, to evaluate the error rate for calls ≥10 ms, which both methods can detect, we repeated the analysis after omitting the short elements from the manual gold standard (Fig 2). For this second error estimate, five files with < 50 manually detected elements were excluded, as larger files likely have more reliable percentages of error rates. Thus, this second analysis provides estimates of error rates for USV detection within the threshold, and it is based on files containing the largest number of calls.

Results are reported as mean ±1 standard deviation, and statistical analyses were conducted in IBM SPSS Statistics 22. To analyze amount of USV emission, we used the total number of elements detected per file (‘number of elements’). We performed non-parametric statistics as the assumptions of parametric statistics were not met, used two-tailed tests, and results are considered statistically significant at α ≤ 0.05.

### Ethical statement

This study was carried out in strict accordance with the recommendations in the Guide for the Care and Use of Laboratory Animals of the National Institutes of Health. All the experiments were conducted at the Konrad Lorenz Institute of Ethology, Austria and the protocols have been approved and were in accordance with ethical standards and guidelines in the care and use of experimental animals of the Ethical and Animal Welfare Commission of the University of Veterinary Medicine Vienna (Austria).

## Results

We first confirmed the reliability of our gold standard, and we compared manual segmentation results between observers, who both visualized spectrograms using the same program (STx), and between different programs, evaluated by the same observer. The number of elements detected was highly correlated between observers and between programs (Spearman’s rho, n = 10, ρ = 0.99, p < 0.001 for both correlations). Thus, manual detection was highly repeatable regardless of the observer or the software used for visualizing spectrograms, suggesting that any potential influence of different parameters used to generate the spectrograms within the two programs is negligible. We then used our generated gold standard (Nref) to calculate and compare error rates of A-MUD with a widely used commercial software. First, we calculated the proportion of correct positives, false positive, and false negative elements (candidate syllables; see Methods) for both A-MUD and the commercially available program (with the two different settings) (Fig 1). We found that A-MUD was significantly more reliable for detecting (a) correct positives (A-MUD vs. commercial software, whistle tracking: Wilcoxon signed ranks test, Z = -2.8, n = 10, p = 0.005 and A-MUD vs. commercial software, single threshold: Wilcoxon signed ranks test, Z = -2.8, n = 10, p = 0.005; Fig 1A); (b) avoiding false positives (A-MUD vs. commercial software, whistle tracking: Wilcoxon signed ranks test, Z = -2.8, n = 10, p = 0.005 and A-MUD vs. commercial software, single threshold: Wilcoxon signed ranks test, Z = -2.8, n = 10, p = 0.005; Fig 1B); and (c) avoiding false negatives (A-MUD vs. commercial software, whistle tracking: Wilcoxon signed ranks test, Z = -2.8, n = 10, p = 0.005 and A-MUD vs. commercial software, single threshold: Wilcoxon signed ranks test, Z = -0.56, n = 10, p = 0.58; Fig 1C). Thus, A-MUD had lower error rates than the commercial software for most comparisons.

Second, we re-calculated error rates after removing the short elements (<10 ms) from the gold standard, and excluding small files with only few (<50) manually detected elements. Again, we found that A-MUD was significantly more reliable than the commercial software (Fig 2) for most comparisons: (a) correct positives (A-MUD vs. commercial software, whistle tracking: Wilcoxon signed ranks test, Z = -2.02, n = 5, p = 0.04 and A-MUD vs. commercial software, single threshold: Wilcoxon signed ranks test, Z = -2.02, n = 5, p = 0.04; Fig 2A); (b) false positives (A-MUD vs. commercial software, whistle tracking: Wilcoxon signed ranks test, Z = -2.02, n = 5, p = 0.04 and A-MUD vs. commercial software, single threshold: Wilcoxon signed ranks test, Z = -2.02, n = 5, p = 0.04; Fig 2B); and (c) false negatives (A-MUD vs. commercial software, whistle tracking: Wilcoxon signed ranks test, Z = -2.02, n = 5, p = 0.04 and A-MUD vs. commercial software, single threshold: Wilcoxon signed ranks test, Z = -0.14, n = 5, p = 0.89; Fig 2C).

Thus, A-MUD had significantly lower error rates than the commercial software in both approaches of evaluation. The second method arguably provides the more informative assessment of A-MUD and the commercial software for calls ≥10 ms, as it excluded sounds below the defined threshold, which is a known constraint. The results indicate that A-MUD had more correct positives, fewer false positives, and fewer false negatives compared than the commercial software (Fig 2). The errors for A-MUD were mainly false negatives (mean: 23±16%), rather than false positives (10±6%).

## Discussion

Our aims were to develop an algorithm for automatically detecting mouse USVs, evaluate its performance using manual segmentation (gold standard), and compare error rates with a commercially available program. The main challenge for developing automatic USV detection methods, as for any signal detection task, is to minimize false positives from background noise. We developed an algorithm for automatic USV detection (A-MUD 1.0), and in this validation, we set a threshold so that it would not detect sounds with a very short (<10 ms) duration. This approach probably reduces false positives, but misses very short vocalizations. We considered this to be an acceptable trade-off since most USVs are ≥10 ms, and the problematic background noise is often <10 ms. We recorded USVs of wild-derived mice, and used these recording to evaluate the performance of A-MUD and one commercially available software program. We evaluated error rates using manual segmentation (gold standard reference), which we confirmed to be highly repeatable (between observers and different programs used for manual segmentation), and then we compared the error rates of A-MUD and the commercial program using the same 10 sound files. We first estimated error rates using all of the candidate syllables (elements) in the manual segmentation. However, since short elements in the manual reference were not detected by A-MUD (or the commercial software) due to the threshold, we also evaluated error rates for elements ≥10 ms. Thus, in our second evaluation, we removed short elements from the manual segmentation files, and in addition, we only used files with a large number of USVs (>50 manually detected elements). Again, we found that A-MUD was significantly more reliable for detecting USV elements (correct positives, i.e. confirmed syllables) and also for avoiding false positives and false negatives compared to the commercial software (tested with two different settings) (Figs 1 and 2). Thus, both evaluation methods are consistent and lead to the same conclusion. Errors in A-MUD were mostly false negatives (mean±SD: 23±16%) rather than false positives (10±6%). The false negatives can mainly be attributed to short elements, which can be reduced by increasing the overlap (reducing the hop size), but this change would also increase the computational effort. In addition, we found that low amplitude elements (with a low signal-to-noise-ratio) were often not detected (false negatives).

As expected, A-MUD greatly improved the efficiency of processing USV sound files (i.e., the processing speed was 4–12 times faster than manual segmentation, depending upon the size of the file). A-MUD required ca. 3.5 h for processing all 14 sound files used to develop and validate it, whereas manual segmentation required ca. 30 h. For processing each 10 min recording, A-MUD required 12–15 min per file (the duration depends on the PC processor speed and we used an Intel® Core™ i5-3470 Processor and 8 GB of RAM), whereas manual segmentation required 1–3 h per file, depending on the number of syllables.

Thus, A-MUD provides a fast and reliable method for processing USV data, and it outperformed at least one other method, which is often used in USV studies. More such comparisons of methods would be highly valuable for the field, though it would require publishing the codes and other information necessary for the exact re-implementation (e.g., exact window information for the sonogram, noise thresholds, filtering methods, etc.). We are making this first version of A-MUD available for the scientific community (non-commercial use) at https://www.kfs.oeaw.ac.at/doc/amud/AMUD1b.sts (Script); Readme: https://www.kfs.oeaw.ac.at/doc/amud/AMUD1b-Readme.odt. This link also provides the code for A-MUD 1.0. Additional comparisons, such as analyzing A-MUD with sound files generated by other mice or under different recording conditions are now more feasible with the recent development of an online database for uploading mouse vocalizations (*mouseTube*) [58]. It is unclear whether the error rates we observed in our study will apply to other mice or conditions. Reliable automatic USV detection is especially challenging for recording animals during direct interactions due to the increased background noise from activity. The main challenge is to develop simple and accurate techniques that make it possible to distinguish and identify an individual’s USVs during social interactions [16]. Under such conditions, we find that automatic call detection is error-prone. We are currently developing an improved version of A-MUD to reliably detect and classify syllables when mice are physically interacting.

## Supporting information

S1 Table Data: Automatic Mouse Ultrasound Detector (A-MUD). (XLSX)
